# Oxidative stress-mediated NFκB phosphorylation upregulates p62/SQSTM1 and promotes retinal pigmented epithelial cell survival through increased autophagy

**DOI:** 10.1371/journal.pone.0171940

**Published:** 2017-02-21

**Authors:** Chunjuan Song, Sayak K. Mitter, Xiaoping Qi, Eleni Beli, Haripriya V. Rao, Jindong Ding, Colin S. Ip, Hongmei Gu, Debra Akin, William A. Dunn, Catherine Bowes Rickman, Alfred S. Lewin, Maria B. Grant, Michael E. Boulton

**Affiliations:** 1 Department of Anatomy and Cell Biology, University of Florida, Gainesville, Florida, United States of America; 2 Department of Ophthalmology, Indiana University School of Medicine, Indianapolis, Indiana, United States of America; 3 Departments of Ophthalmology and Cell Biology, Duke University Medical Center, Durham, North Carolina, United States of America; 4 Department of Molecular Genetics and Microbiology, University of Florida, Gainesville, Florida, United States of America; Indian Institute of Science Education and Research, INDIA

## Abstract

p62 is a scaffolding adaptor implicated in the clearance of protein aggregates by autophagy. Reactive oxygen species (ROS) can either stimulate or inhibit NFκB-mediated gene expression influencing cellular fate. We studied the effect of hydrogen peroxide (H_2_O_2_)-mediated oxidative stress and NFκB signaling on p62 expression in the retinal pigment epithelium (RPE) and investigated its role in regulation of autophagy and RPE survival against oxidative damage. Cultured human RPE cell line ARPE-19 and primary human adult and fetal RPE cells were exposed to H_2_O_2_-induced oxidative stress. The human apolipoprotein E4 targeted-replacement (*APOE4)* mouse model of AMD was used to study expression of p62 and other autophagy proteins in the retina. p62, NFκB p65 (total, phosphorylated, nuclear and cytoplasmic) and ATG10 expression was assessed by mRNA and protein analyses. Cellular ROS and mitochondrial superoxide were measured by CM-H2DCFDA and MitoSOX staining respectively. Mitochondrial viability was determined using MTT activity. qPCR-array system was used to investigate autophagic genes affected by p62. Nuclear and cytoplasmic levels of NFκB p65 were evaluated after cellular fractionation by Western blotting. We report that p62 is up-regulated in RPE cells under H_2_O_2_-induced oxidative stress and promotes autophagic activity. Depletion of endogenous p62 reduces autophagy by downregulation of ATG10 rendering RPE more susceptible to oxidative damage. NFκB p65 phosphorylation at Ser-536 was found to be critical for p62 upregulation in response to oxidative stress. Proteasome inhibition by H2O2 causes p62-NFκB signaling as antioxidant pre-treatment reversed p62 expression and p65 phosphorylation when RPE was challenged by H2O2 but not when by Lactacystin. p62 protein but not RNA levels are elevated in *APOE4*-HFC AMD mouse model, suggesting reduction of autophagic flux in disease conditions. Our findings suggest that p62 is necessary for RPE cytoprotection under oxidative stress and functions, in part, by modulating ATG10 expression. NFκB p65 activity may be a critical upstream initiator of p62 expression in RPE cells under oxidative stress.

## Introduction

The ubiquitin-proteasome system (UPS) and the autophagy-lysosome pathway (ALP) comprise two major pathways for the removal and clearance of proteins and damaged organelles in eukaryotic cells. While the UPS is generally believed to degrade short-lived proteins or small protein aggregates [1], the ALP is responsible for clearance of long-lived proteins, bulk protein aggregates and is the only system with the capacity to degrade entire organelles [2]. Numerous age-related neurodegenerative disorders have been associated with dysregulated autophagy [3–5]. Age-related macular degeneration (AMD) is the leading cause of visual loss in the elderly population. Although the pathogenesis of AMD remains unclear there is strong evidence that oxidative stress and impaired protein degradation and clearance pathways in retinal pigment epithelial (RPE) cells may lead to RPE damage and dysfunction [6–10].

Autophagy is crucial in RPE homeostasis because the RPE has high metabolic activity and is exposed to a highly oxidative environment. Therefore, insufficient removal of oxidatively damaged intracellular organelles or protein aggregates due to impaired autophagy or lysosome activity will contribute to intracellular (lipofuscin) and extracellular (drusen) accumulation of toxic materials that impair RPE function [11, 12]. In addition, it has been reported that proteasome activity is reduced in RPE cells during aging or under oxidative stress [13, 14]. It remains unclear how autophagy is activated under oxidative stress in RPE cells and which signaling factors and pathways are involved.

p62/ Sequestosome1 (SQSTM1) is a multi-functional scaffold protein playing critical roles in various cell signaling pathways and working as an adaptor molecule in different cellular processes (16–21). In particular, p62 contains a ubiquitin-associated domain (UBA) and a microtubule-associated protein 1 light-chain 3 (LC3)-interacting region (LIR), which acts as a receptor that binds and delivers polyubiquitinated proteins to the UPS or to ALP for degradation when proteasomes are impaired or overwhelmed [15]. This suggests that p62 is a critical link between the proteasomal and lysosomal clearance systems [16, 17]. It has recently been demonstrated that proteasome inhibition caused elevated p62 expression in RPE cells [18]. The p62 promoter contains an antioxidant response element (ARE) and is up-regulated by oxidative stress via the transcription factor NF-E2-related factor 2 (Nrf2) [19]. In turn, p62 binds to KEAP1 and thus stabilizes Nrf2 leading to a positive feedback loop for antioxidant gene expression [20]. Reports in other cell and tissue types underline a critical interplay between p62, the NFκB signaling pathway and autophagy [21–25]. Although, the interplay between autophagy and NFκB is cell type- and trigger-dependent, oxidative stress has been shown to upregulate autophagy via activation of NFκB in senescent keratinocytes and MCF-7 epithelial cancer cell lines [21, 25] with the suggestion that NFκB can act as a direct transcriptional mediator of p62 expression [26]. Although NFκB signaling has been studied in the RPE in the context of pro-inflammatory signaling [27, 28], its role in p62 regulation is unexplored.

In this study, we show that exposing RPE cells to oxidative stress results in the inhibition of proteasome activity and a dramatic increase in p62 gene expression, which positively regulates the autophagy clearance pathway. These data support the hypothesis that proteasome inhibition by oxidative stress is a mechanistic trigger for p62 upregulation and autophagy activation. Using a combination of pharmacological IKKβ inhibitor, NFκB p65 S536D/S536A dominant phosphorylation positive/negative mutant and competing short peptide we demonstrated that NFκB p65 phosphorylation at Serine 536 is a critical step in p62 upregulation in RPE under oxidative stress. Moreover, we show that knockdown of p62 attenuates H_2_O_2_-induced autophagy and makes RPE cells more susceptible to oxidative stress. p62, at least in part, regulates the expression of ATG10, a processing enzyme that facilitates formation of the autophagosomal precursor. Our study reveals that oxidative stress-induced p62 expression stimulates autophagy, which serves as an early protective response in RPE cells. Attenuation of endogenous ATG10 increases the susceptibility of RPE to oxidative stress.

## Material and methods

### Cell culture and treatment paradigm

The human ARPE-19 cell line obtained from American Type Culture Collection (Cat# CRL-2302, ATCC, Manassas, VA) [29] was grown in Ham's F10 Medium (Cat# 10-070-CV, Mediatech, Manassas VA) containing 10% fetal bovine serum (FBS) (Cat# 12107C, Sigma Aldrich, St. Louis, MO) to a confluent monolayer at 37°C in a humidified atmosphere containing 5% CO_2_ (9). Cells were used within passage number 25 (i.e. within 6 passages after receipt from ATCC). Commercially available human primary fetal RPE (Cat# 00194987) was obtained from Lonza Group (Basel, Switzerland) and cultured in RtEGM™ (Cat# 00195409, Lonza) Retinal Pigment Epithelial Cell Growth Medium as per manufacturer’s instructions.

For experiments, ARPE-19 cells were maintained at confluence and the medium was replaced with Ham’s F10 + 2% FBS 48 hours before experimental procedures. Primary fetal RPE growth media was replaced with basal media (containing 0.8% serum) 24 hours before experimental procedure. RPE cells were exposed to sublethal doses of H_2_O_2_ (Cat# 216763, Sigma-Aldrich, St. Louis, MO) as previously described [30] with or without p62 or ATG10 siRNA or vector for the indicated duration of the respective experiments. Untreated or vehicle-treated cells were used as controls. Human recombinant Tumor Necrosis Factor-α (TNF-α) at final concentration 10ng/ml (Cat# T0157, Sigma Aldrich, St. Louis, MO) was used as positive control for NFκB activity in ARPE-19 cells. IKKβ pharmacological inhibitor SC-514 (Cat# S4907) at final concentration 100μM was obtained from Selleckchem (Houston, TX). For studies involving antioxidants, ARPE-19 cells were either treated with N-acetylcysteine amide (NACA) (Sigma Aldrich, Cat# A0737) (1mM for 1 hour) or MitoTEMPO (Sigma Aldrich, Cat# A0737) (250μM for 1 hour) prior to challenge with either H_2_O_2_ or Lactacystin for 6 and 12 hours respectively.

### Animals, diet and treatment protocols

We used human apolipoprotein E4 targeted-replacement (*APOE4)* mice, which at the age of 16 months were switched from a normal rodent diet (ND) (Isopurina 5001; Prolab, Dewitt, NY) to a high fat, cholesterol (HFC) enriched diet (TD 88051; Harlan Teklad, Madison, WI) for 2 months as described in Malek *et al*., 2005 [31]. Age-matched *APOE4* mice continuously maintained on a ND (*APOE4-*ND) were used as a control group to compare to the *APOE4*-HFC mice (n = 6 or 10 per group as indicated in figure legends). Eyes were prepared for immunohistochemistry or dissected and the RPE/choroid layers separated and lysed in RIPA (25mM Tris-HCl (pH 7.6), 150mM NaCl, 1% NP-40, 1% sodium deoxycholate, 0.1% SDS) buffer containing protease inhibitor to extract protein lysate for Western blotting. Separately, isolated retinas were also put in RNA-later for processing of RNA for quantitative Real-Time RT-PCR. All animal procedures were in agreement with the ARVO Statement for the Use of Animals in Ophthalmic and Visual Research and in compliance with Institutional Animal Care and Use Committee (IACUC) guidelines. Duke University IACUC approved this study: Protocol #A162-12-06.

### Immunofluorescence

Immunocytochemical staining was used to detect p62 expression and its association with autophagosomes. After transfection and treatment, ARPE-19 cells were fixed in 4% paraformaldehyde. Cells were washed three times with phosphate buffered saline (PBS), permeabilized and blocked with blocking buffer (1% bovine serum albumin, 5% goat serum in PBS), containing 0.2% Triton X-100 in PBS for 1h. Cells were then incubated overnight with primary antibody against p62 (Cat# 610832, BD Biosciences, San Jose, CA). Alexa594®-conjugated anti-mouse antibody was used as secondary antibody (Life Technology, Carlsbad, CA). Nuclei were counterstained with Vectashield DAPI (H-1200) mounting medium (Vector Labs, Burlingame, CA) and analyzed by confocal microscopy.

Retinal sections of the AMD mouse (*APOE4-*HFC) model and control (*APOE4*-ND) were prepared by standard paraffin embedding. 4μm thick sections were cut and air-dried. Sections were then deparaffinized/rehydrated in xylene and serial dilutions of ethanol followed by antigen unmasking with Rodent Decloaker (Biocare Medical, CA) and were blocked with 5% normal goat serum and 1% BSA for 1h at room temperature. Mouse monoclonal anti-p62 was diluted (1:50) in PBS with 1% BSA and incubated for 2h at room temperature. After washing with PBS, the sections were incubated with secondary antibodies conjugated with tetramethylrhodamine (TRITC) for 1h at room temperature in the dark. Sections were covered with Vectashield® mounting medium and photographed.

### Western blot analysis

RPE cells or mouse RPE/choroid tissue were lysed in RIPA buffer containing protease inhibitor. Cytosolic and nuclear fractions of cells lysates were purified using the NE-PER™ nuclear and cytoplasmic extraction reagent (Thermo Fisher, Waltham, MA) following the manufacturer’s guidelines. Cell lysates containing equal amounts of protein were loaded and separated on a 4–20% gradient SDS-PAGE gel and transferred to PVDF membrane. After blocking nonspecific binding sites with Licor blocking buffer for 1h at room temperature membranes were incubated overnight with primary antibodies against LC3 (Cat# NB100-2220), ATG10 (Cat# NBP2-38524) (dilution 1:1000) (Novus Biologicals, Littleton, CO), or p62 (1:2000) at 4°C. The primary antibody treatments were followed by treatment with secondary IR dye-800 conjugated anti-rabbit dye or AlexaFlour680® conjugated anti-mouse IgG for 1h at room temperature. β-actin (1:5000 antibody dilution) (Cat# A5441, Sigma Aldrich, St. Louis, MO) was used as loading control. Western blot images were captured with the Odyssey CLx® infrared imaging system (LI-COR Biosciences, Lincoln, NE) and analyzed for densitometric analysis using Odyssey 2.0 software.

### GFP-LC3 overexpression and puncta detection

ARPE-19 cells were transiently transfected with GFP-LC3 with or without p62 siRNA transfection by the Lipofectamine LTX® reagent (Invitrogen, Carlsbad, CA) following manufacturer’s guidelines. After 24 h, the cells were treated with H_2_O_2_ and fluorescent microphotographs of GFP-LC3 were obtained using a fluorescence microscope (Zeiss HBO 100) and analyzed by Axiovision Rel 4.4 software (Zeiss, Oberkochen, Germany). Detection of GFP-LC3 positive punctae following oxidative stress was used to quantify autophagosome formation. Mean autophagosome counts from 50 cells/treatment were obtained from three separate experiments.

### Quantitative real time PCR

After respective treatments, RNA was extracted from cells by TRIzol reagent (Invitrogen, Carlsbad, CA), quantified by Nanodrop^TM^ 2000c UV-Vis Spectrophotometer (Thermo Scientific, Waltham, MA) and 1μg RNA was reverse transcribed into cDNA with random primers using AffinityScript^TM^ qPCR cDNA Synthesis Kit (Agilent Technologies, Santa Clara, CA) (20μl reactions). PCR reactions were amplified on a Bio-rad CFX-96 real-time PCR system using the SsoAdvanced Universal SYBR Green Supermix® (Bio-rad, Berkley CA). GAPDH was used as an internal control. All primers were obtained from the Prime PCR Assay catalog of pre-validated qRT-PCR primers. Real Time PCR data was analyzed using the 2^-ΔΔC^_T_ method (38).

For the *APOE4* mouse samples, RNA was extracted from the RPE/choroid lysates using a combination of TRIzol reagent and Qiagen RNeasy Mini kit. 500ng of total RNA was reverse transcribed to cDNA using the iScript^TM^ Reverse Transcriptase kit (Bio-rad, Berkley, CA) (20μl reactions). Further downstream steps for qRT-PCR were the same as described above.

### Proteasomal peptidase assay

Proteasomal peptidase assay was performed as described previously (39). Briefly, after treatment RPE cells were harvested and lysed in hypotonic buffer (10mM HEPES, 5mM MgCl_2_, 10mM KCl, 1% sucrose, and 0.1% CHAPS). Lysates were then incubated with fluorogenic substrate Suc-LLVY-AMC (75μM) in the assay buffer (50mM Tris-HCl, 20mM KCl, 5mM MgOAc and 10mM dithiothreitol, pH 7.6) for 30 min at 37°C. Cleaved fluorescent products were then examined at the excitation wavelength of 380 nm and emission wavelength of 460 nm by a fluorescence plate reader (Biotek, Winooski, VT). Enzymatic activities were normalized by protein concentration, which was measured using the Bradford method.

### Transfection experiments

ARPE-19 cells were transfected by Lipofectamine® RNAiMAX with duplex oligoribonucleotides against p62/SQSTM1 or with scrambled siRNA (Silencer® Select siRNA, Life Technologies, Carlsbad, CA) for 24 h. Cells were then treated with 400μM H_2_O_2_ as described above. For ATG10 knockdown experiments ARPE-19 cells were transfected with either scrambled or Flexitube ^®^ siRNA oligos from Qiagen.

For overexpression experiments, ARPE-19 cells were transfected with a p62 construct (pTR-SC-smCBA-p62/SQSTM1) or empty vector (pTR-SC-smCBA) using Lipofectamine^®^ LTX reagent. Similarly, p65 wild type (p65-WT), p65 dominant positive mutant serine to aspartic acid (p65-S536D) and p65 phosphorylation negative mutant serine to alanine (p65-S536A) on vector pcDNA3.1 were transfected in ARPE-19 cells for the NFκB p65 overexpression experiments.

### Cellular reactive oxygen species (ROS) and superoxide detection

CM-H2DCFDA (Thermo Scientific, Cat# C6827) and MitoSOX™ Red (Invitrogen, Cat # M36008) were used to detect cellular ROS and mitochondrial superoxide formation respectively in ARPE-19 cells following stress procedures. After incubating cells with or without H_2_O_2_ (6 Hrs for CM-H2DCFDA and 1 Hrs for MitoSOX™) or Lactacystin (12 Hrs) on black, clear-bottom cell culture treated 96-well tissue culture plate (Corning^TM^, Cat # 3603), cells were loaded with CM-H2DCFDA (10 μM) or Mitosox (2μM) dye for 10 minutes at 37°C. Following the incubation, the plate was first centrifuged at 200g at room temperature and then the cells were gently washed in PBS (1X) once and immediately analyzed on a Biotek® Fluorescence Microplate Reader (CM-H2DCFDA = excitation/emission: 492/527 nm and MitoSOX™ Red = excitation/emission: 510/580 nm).

### MTT Assay

3-(4, 5-Dimethylthiazol-3-yl)-2, 5-diphenyl Tetrazolium Bromide (MTT) assay (Cat# M5655, Sigma Aldrich, St. Louis, MO) was used to assess mitochondrial activity after H_2_O_2_ treatment with or without p62 siRNA transfection. Briefly, after treatment ARPE-19 cells were washed once and then incubated with 0.25mg/ml MTT (Sigma Aldrich, St. Louis, MO) in serum-free medium at 37°C for 3 h. The medium was then removed and dimethyl sulfoxide was added to solubilize the produced blue formazan (MTT metabolic product). The density of blue formazan was further measured at 570 nm with a reference wavelength at 630nm using a microplate reader.

### Cell viability by crystal violet assay

The cell viability was determined by crystal violet uptake as described in our previous publication [32]. Briefly, after treatment, cells were fixed in 4% paraformaldehyde in PBS for 15 minutes and stained in a solution of 0.1% crystal violet (Sigma Aldrich, St. Louis, MO), 10% ethanol for 5 minutes. After washing three times with PBS, the plates were air-dried and the remaining stain was dissolved in, 10% acetic acid and absorbance measured with a microplate reader at 540 nm.

### Measurement of cell numbers

Cell numbers were measured by trypsinization of adherent cells and centrifugation at 200g for 3 minutes. The pellet was resuspended in 500μl media. The cells were counted in the TC10® cell counter (Bio-rad) expressed as number of cells/ml.

### Determination of Trans-epithelial Resistance (TEER)

TEER measurements were performed using an EVOM2 volt-ohmmeter and EndOhm chamber (World Precision Instruments, Sarasota, FL) as previously described [33]. Briefly, cells were seeded on 24-well plate cell culture inserts (0.4μM pore size, Transwell, Corning, Cambridge, MA) with 0.5μl media in both chambers. Resistance readings (Ω) were obtained every 24 hours for two weeks by placing each insert in the EndOhm chamber connected to the EVOM2 meter. The resistance value of an empty culture insert (no cells) was subtracted.

### Statistics

Data were analyzed using Student’s t-test, one-way ANOVA, or Mann-Whitney for comparisons wherever appropriate. Differences between means from at least 3 independent experiments with p<0.05 were considered statistically significant. Analyses were performed using Prism 5.0 software (GraphPad Software, San Diego, CA).

## Results

### Oxidative stress increases autophagic activity and p62 expression in RPE cells

**Fig 1:**
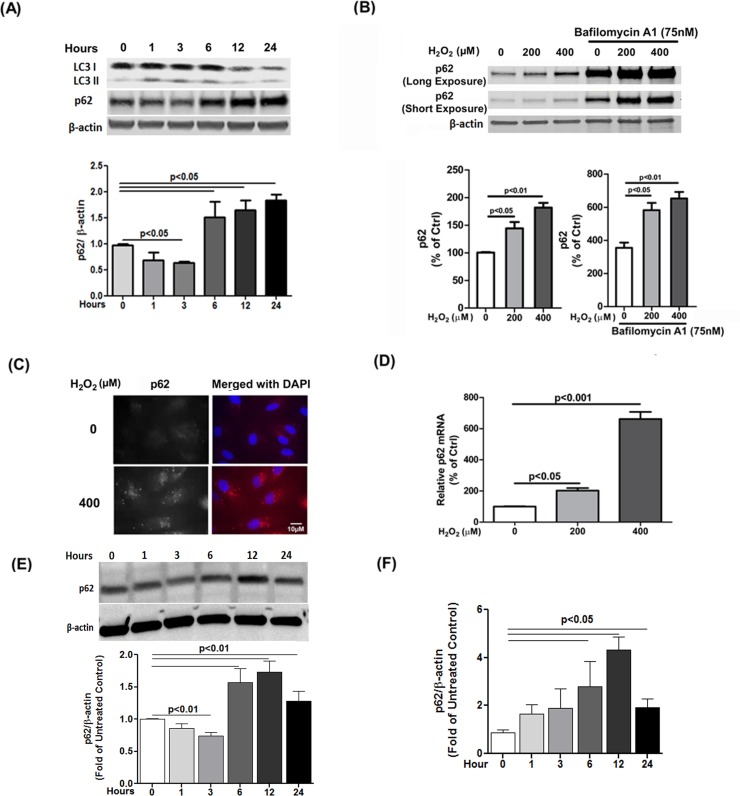
H_2_O_2_ exposure increases autophagic flux and p62 expression in RPE cells. **(A)** ARPE-19 cells were exposed to 400μM H_2_O_2_ and autophagy was monitored on western blots of protein lysates following treatment at 1, 3, 6, 12 and 24h by LC3 II/I conversion and p62/SQSTM1 levels using anti-LC3 and anti-p62/SQSTM1 antibodies respectively. β-actin was used as an internal control (upper panel). p62 levels were analyzed by densitometry as described in Experimental Procedures (lower panel). **(B)** ARPE-19 cells were exposed to 200 or 400μM H_2_O_2_ for 24h and p62 protein levels were monitored by Western blot and analyzed by densitometry. β-actin was used as an internal control. Control (Ctrl) is H_2_O_2_ (0μM) without Bafilomycin A1. ‘Short’ and ‘Long’ Exposure indicate time of exposure of immunoblot image. **(C)** Confocal images of control ARPE-19 cell cultures and ARPE-19 cells following 6h exposure to 400μM H_2_O_2_ labelled with anti-p62 (red) and nuclear-stained with DAPI (blue). **(D)** ARPE-19 cells were treated with 200 or 400μM H_2_O_2_ for 24h and mRNA extracted. p62 mRNA level was determined by qRT-PCR with p62 specific primers. **(E)** Primary human fetal RPE cells were exposed to H_2_O_2_ (400μM) and and p62/SQSTM1 levels were monitored by Western blot of protein lysates following treatment at 1, 3, 6, 12 and 24h using anti-p62 antibody. β-actin was used as an internal control (upper panel). Densitometric analyses were plotted in the lower panel. **(F)** Primary human fetal RPE cells were treated with H_2_O_2_ for 24h and then p62 mRNA level was examined by relative quantitative real-time qRT-PCR with p62 specific primers. Statistical significance between untreated control and H_2_O_2_-exposed cells was determined by Mann-Whitney U-test, p < 0.05.

We have previously reported that acute sublethal dose of H_2_O_2_ increases autophagy in RPE cells by measuring the lipidation of microtubule-associated protein 1A/1B-light chain 3 (LC3) as well as autophagosome numbers by LC3 immunostaining (9). In addition to a steady increase in LC3II/I ratio, we observed an initial reduction of p62 (a marker for autophagic activity) at 3h after oxidative stress but a subsequent increase in levels over a 24h time-course (Fig 1A). Furthermore, co-treatment with 75nM Bafilomycin A1, which blocks the fusion of autophagosomes with lysosomes, did not block H_2_O_2_-induced p62 accumulation thus confirming that the increase in p62 protein levels is not due to autophagy deficiency (Fig 1B). To verify the Western blot data we also observed a dramatic increase of p62 staining in H_2_O_2_-treated cells compared to controls by immunocytochemistry (Fig 1C). Incidentally, induction of p62 gene expression has been previously reported in SH-SY5Y neuroblastoma cells at 24h after oxidative insult by H_2_O_2_ [32], suggesting that the increase in p62 in our system could be due to increase in gene expression. We therefore confirmed that p62 gene expression was increased in a dose-dependent fashion following exposure of RPE cells to oxidative stress compared to untreated control (Fig 1D). It is possible that increased p62 levels under oxidative stress are a result of transcription-induction mediated by NRF2 [19]. To confirm that our observations were not a feature of the ARPE-19 cell line used we further assessed p62 expression and autophagic flux in human primary fetal RPE cultures. As previously reported [34], these RPE cells showed greater TEER indicating stronger barrier function than ARPE-19 cells (data not shown). p62 expression both at protein and mRNA levels peaked at 12 h after H_2_O_2_ (200μM) challenge (Fig 1E and 1F). Collectively, the data demonstrate that autophagic flux increases under oxidative stress, which is associated with an increase in p62 expression in both primary RPE cells as well as in the ARPE-19 cell line.

### p62 is increased in the retina of AMD mouse model

**Fig 2:**
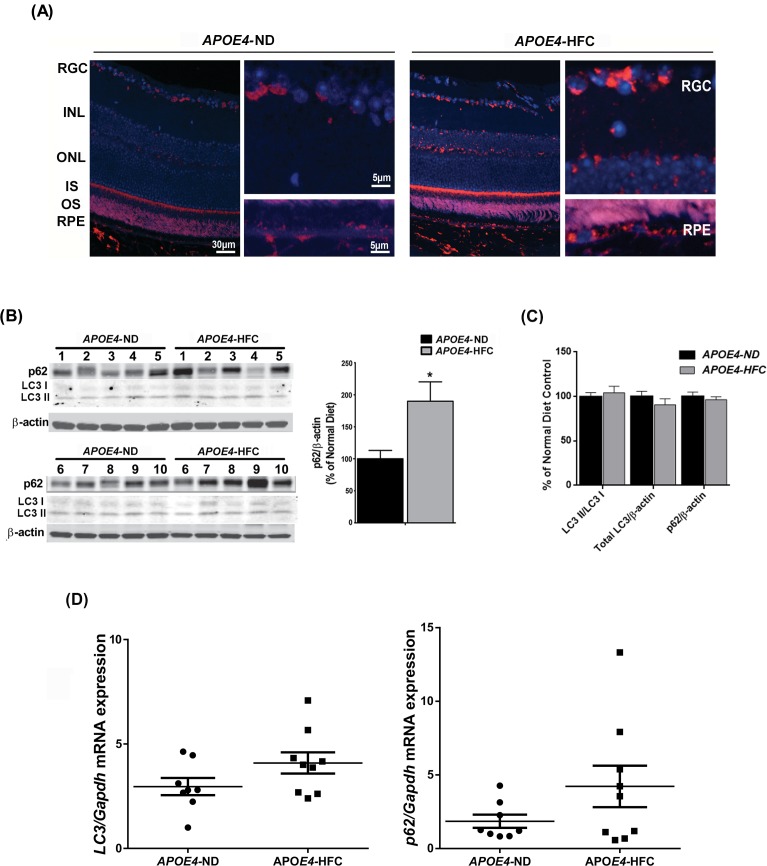
p62 expression is enhanced in AMD mice model. APOE4 mice at the age of 18–20 months were fed a high fat, cholesterol enriched diet (HFC) for 2 months and control age-matched APOE4 mice were kept on a normal diet (ND), (**A)** Paraffin embedded mouse retina sections from each group (n = 6, disease and control groups) were incubated with monoclonal p62 antibody followed by secondary antibodies conjugated with AlexaFluor® 594 dye. Sections were covered with Vectashield mounting medium/DAPI. Photographs were taken by Zeiss AX10.1 Observer fluorescent microscope. **(B)** Protein lysates from RPE/Choroid layers (pooled left and right eye cup for each mouse) were analyzed by Western blot against p62 and LC3. 10 mice were used per group. β-actin was used as an internal control. Lane numbers denote the animal number in each group. Densitometric quantification of p62 band intensity was analyzed. Statistical significance between the normal diet (n = 10) and high-fat diet-fed groups (n = 10) was determined by ANOVA, p<0.05. **(C)** Protein lysates from the neural retina (pooled left and right retina for each mouse) were analyzed by Western blot against p62 and LC3. 10 mice were used per group. β-actin was used as an internal control. Densitometric quantification of p62, LC3 (II/I) ratio and total LC3 were plotted. Statistical significance between the normal diet (n = 10) and high-fat diet-fed groups (n = 10) was determined by ANOVA, p<0.05. **(D)** Real Time PCR analyses for Lc3 and p62 in mouse RPE/Choroid was performed with Gapdh as internal control.

Since AMD is a disease that is associated with oxidative injury to the RPE [35] we next determined if our *in vitro* findings could be recapitulated in the *APOE4*-HFC diet mouse model of AMD [31]. Immunostaining of p62 was greatly increased in RPE and choroid regions of *APOE4*-HFC mice compared to control animals (Fig 2A). Western blot analyses demonstrated significant increases in p62 levels in the RPE/choroid tissue lysates (Fig 2B) but not in the retina lysates of *APOE4*^-^HFC mice (Fig 2C). Furthermore, Western blot demonstrated a small but significant increase in the LC3II/I ratio as shown in Fig 2B and previously reported by Mitter *et al* [30]. Our earlier report on mouse models of AMD (namely, the *APOE4* and superoxide dismutase 2 knockdown models of AMD) showed that levels of autophagic markers LC3, ATG7 and ATG9 were reduced in retina of diseased mice compared to controls suggesting that autophagic efficiency may be compromised in these models of AMD [30]. Increased levels of p62 in these samples suggest that the accumulation of p62 may be a result of reduced autophagy. We tested whether p62 mRNA expression was elevated in the AMD model compared to control but we did not observe significant differences in the p62 mRNA levels between *APOE4*-ND controls and *APOE4*-HFC AMD mouse model RPE/choroid lysates (Fig 2D). We conclude that the elevated p62 in this AMD mouse model may be due to p62 protein accumulation secondary to a reduction of its degradation supporting the hypothesis that autophagic flux is reduced in AMD.

### Oxidative stress increases p62 expression in RPE cells via inhibition of ubiquitin-proteasome function

**Fig 3:**
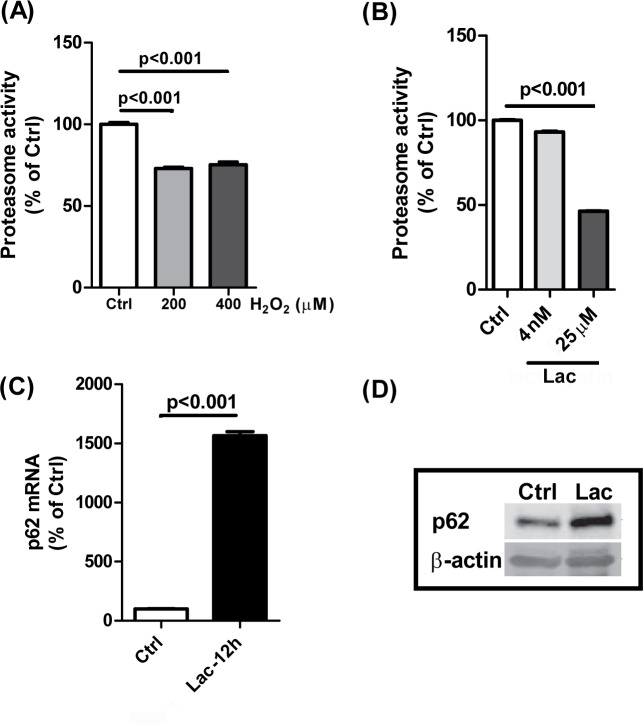
H_2_O_2_ inhibits proteasome activity and proteasome inhibitor lactacystin induces p62 up-regulation. **(A)** ARPE-19 cells were treated with 200 or 400μM H_2_O_2_ and then chymotrypsin-like proteasome activity was measured at 24h post-exposure using a fluorogenic substrate. Control (Ctrl) = No H_2_O_2_ **(B)** Chymotrypsin-like proteasome activity was detected in RPE cells treated with the proteasome specific inhibitor lactacystin (4nM and 25μM, 12h). Ctrl = DMSO control **(C)** qRT-PCR was used to measure the p62 mRNA level following lactacystin treatment (25μM, 12h). Ctrl = DMSO control **(D)** p62 protein level was examined by western blot after proteasome specific inhibitor lactacystin (25μM, 12h) exposure. Ctrl = DMSO control. Data represent the mean + S.E.M. for three samples per group. Differences in means were considered statistically significant when p<0.05.

Known as a “shuttling factor”, p62 delivers ubiquitinated substrates to the proteasome (UPS pathway) or lysosomes (ALP) for degradation [36]. Through measurement of proteasomal peptidase activity, we determined that H_2_O_2_ exposure significantly inhibited proteasome activity in ARPE-19 cells within 24h (Fig 3A). Treatment of ARPE-19 cells with the proteasome specific inhibitor Lactacystin reduced proteasome activity by as much as 50% (Fig 3B) and induced a dramatic increase in both p62 gene and protein expression (Fig 3C and 3D), indicating that proteasome inhibition might be responsible for triggering p62 expression under oxidative stress. NRF2 may play a critical role since it shuttles to the nucleus and induces p62 expression under proteosomal and oxidative stress [19].

### p62 expression under oxidative stress is dependent on NFκB p65 Ser536 phosphorylation

**Fig 4:**
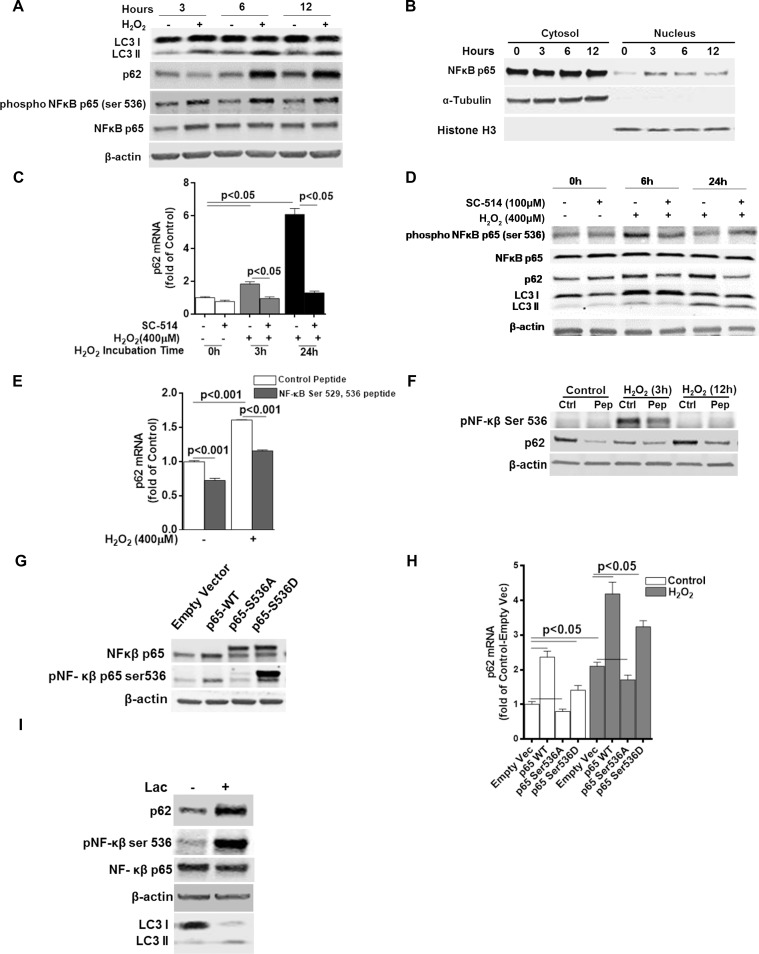
p62 expression under oxidative stress is dependent on NFκB p65 Ser536 phosphorylation. **(A)** ARPE-19 cells were treated with H_2_O_2_ (400μM) for 3, 6 and 12 hours and p62, NFκB p65 ser 536 phosphorylation, and total NFκB p65 protein levels were measured by western blot. β-actin was used as an internal control. Representative western blot from atleast 3 independent experiments has been shown. **(B)** NFκB p65 nuclear translocation was determined after treating ARPE-19 cells with H_2_O_2_ (400μM) for 3, 6 and 12 hours and western blotting on separated nuclear and cytosolic fraction lyastes. Purity of cytosolic and nuclear fractions were determined by blotting for tubulin and Histone (H3) respectively. Representative Western blot from at least 3 independent experiments are shown. **(C)** p62 mRNA levels were measured by qRT-PCR after pretreating the ARPE-19 cells with IKKβ inhibitor SC-514 (100μM) for 1 hour and subsequently co-treating with H_2_O_2_ (400μM) for 3 or 24 hours. **(D)** ARPE-19 cells were pretreated with IKKβ inhibitor SC-514 for 1 hour and subsequently co-treated with H_2_O_2_ (400μM) for 6 or 24 hours. p62, LC3, NFκB p65 ser 536 phosphorylation, and total NFκB p65 protein levels were measured by western blot. β-actin was used as an internal control. Representative Western blot from at least 3 independent experiments is shown. **(E)** ARPE-19 cells were either treated alone or co-treated with competing NFκB Ser529, 536 short peptide or co-treated with H_2_O_2_ (400μM) for 3 hours. p62 mRNA levels were measured by qRT-PCR. **(F)** ARPE-19 cells were either treated alone or co-treated with competing NFκB Ser529, 536 short peptide or co-treated with H_2_O_2_ (400μM) for 3 or 24 hours. p62 and NFκB p65 ser 536 phosphorylation protein levels were measured by western blotting. Ctrl = Untreated control; Pep = NFκB Ser529, 536 short peptide treated. **(G)** Empty pcDNA3.1 vector, p65-WT, dominant negative mutant p65-S536A and dominant positive mutant S536D were transiently overexpressed in ARPE-19 cells for 24 hours and cell lysates were analyzed for NFκB p65 ser 536 phosphorylation and total NFκB p65 protein levels by western blotting. **(H)** ARPE-19 cells transfected with respective plasmids for 24 hours were either left untreated or treated with H_2_O_2_ (400μM) for an additional 3 hours and p62 mRNA levels were measured by qRT-PCR. Data represent the mean +/- S.E.M. for three samples per group. Differences in means were considered statistically significant when p<0.05. **(I)** p62, NFκB p65 ser 536 phosphorylation and total NFκB p65 protein levels were measured by Western blot. β-actin was used as an internal control. Representative western blot from at least 3 independent experiments is shown.

It has been suggested that p62 might act as a scaffold molecule poly-ubiquitinating TRAF6 and thereby constitutively activating IKKβ and NFκB [23]. Additionally, p62 has been suggested to be a direct transcriptional target of NFκB [26]. NFκB signaling plays a critical role in cell inflammatory signaling and survival. We wanted to investigate whether p62 is a transcriptional target of NFκB in the RPE under oxidative stress. ARPE-19 cells showed activation of NFκB p65 (hereafter referred to as NFκB) with a dramatic increase in p65 Serine (536) phosphorylation level up to 12 h after challenge with H_2_O_2_ (Fig 4A). Although a large amount of total cellular NFκB remained cytoplasmic, we observed a significant increase in nuclear NFκB after oxidative challenge after exposure to H_2_O_2_ suggesting that NFκB may play a role in transcriptionally activating its target genes in RPE under oxidative stress (Fig 4B). Pharmacological inhibition of NFκB p65 Serine (536) phosphorylation by SC-514, an ATP dependent competitive inhibitor of IKKβ, significantly reduced p62 mRNA as well as protein levels in ARPE-19 cells under oxidative stress (Fig 4C and 4D). At both 6 and 24 hours, p62 protein levels were dramatically elevated in H_2_O_2_ treated ARPE-19 cells compared to untreated control. NFκB p65 Serine (536) phosphorylation levels at 6 hours in SC-514 treated cells, did not increase in contrast to cells treated with H_2_O_2_ alone suggesting strong inhibition of IKKβ activity and subsequent loss of NFκB p65 activity. However LC3 (II/I) ratios were decreased in SC-514 treated cells compared to respective untreated (no SC-514) controls both with and without oxidative stress (Fig 4D). Furthermore, a short cell permeable NFκB competing peptide containing NFκB ser 529, 536 sites inhibited H_2_O_2_-induced increase of NFκB p65 phosphorylation and p62 expression (Fig 4E and 4F). To further establish NFκB phosphorylation as a critical step for p62 expression, we decided to individually overexpress wild type NFκB (p65-WT), dominant negative mutant (p65-S536A) and dominant positive mutant (p65-S536D) in ARPE-19 cells and observe the effect on p62 expression (Fig 4G). As expected, in untreated (i.e. cells not treated with H_2_O_2_) or H_2_O_2_-treated cells, p65-WT and p65-S536D overexpression resulted in a higher level of p62 mRNA levels than empty vector-transfected cells, while phosphorylation dominant negative p65-S536A transfection showed significant although slight reduction in p62 (Fig 4H). Altogether, data in Fig 4 suggested a critical role of NFκB S536 phosphorylation in H_2_O_2_-induced p62 expression. Activation of NFκB and its nuclear translocation are dependent on its dissociation from the NFκB-IκB complex. IκB is subsequently degraded by the ubiquitin-proteasomal degradation system. Since proteasomal inhibition in the RPE by Lactacystin caused rapid induction of p62 expression, we wanted to determine the effect of proteasomal inhibition on NFκB phosphorylation status. Unexpectedly, 12 hours after incubation with Lactacystin we saw a dramatic increase in the Serine 536 phosphorylation levels with concomitant increase in p62 protein level (Fig 4I). This apparently paradoxical observation may be aluded to the fact that prolonged proteasomal inhibition with Lactacystin has been shown to persistently activate NFκB p65 (see Discussion section) (46).

### Inhibiting cellular reactive oxygen species but not mitochondrial superoxide attenuates p62 expression under oxidative stress

**Fig 5:**
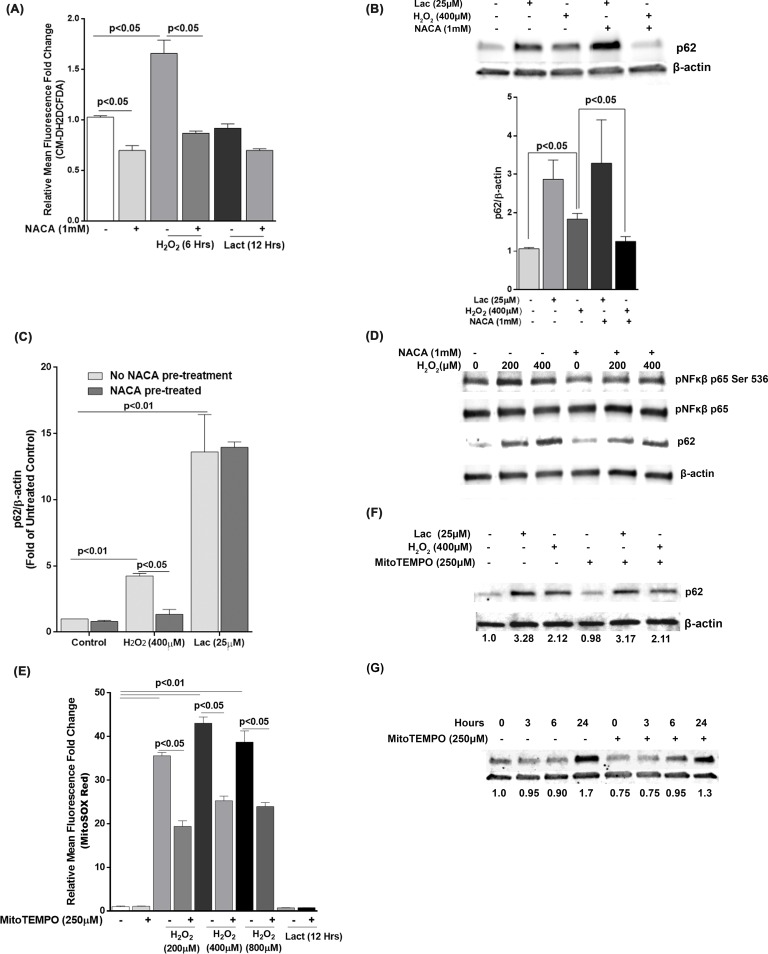
Inhibition of cellular ROS but not mitochondrial superoxide alone can attenuate p62 expression in RPE. **(A)** ARPE-19 cells were pre-treated with, and subsequently challenged with, H_2_O_2_ (400μM) for 6 hours or with Lactacystin (25μM for 12 hours) and cellular ROS was measured by CM-H2DCFDA assay. The mean from 3 independent experiments is plotted as Mean +/- SEM. Differences in means was considered significant when p<0.05 by analysis of variance. **(B)** p62 protein level in ARPE-19 cells after pre-treatment with NACA (1mM) for 1 hour and subsequent challenge with H_2_O_2_ (400μM) for 6 hours or with Lactacystin (25μM for 12 hours) was determined by Western blotting. Representative Western blot from 3 independent experiments are shown. Densitometric data was plotted as Mean +/- SEM for the three experiments. β-actin was used as internal control. **(C)** p62 mRNA levels were measured by qRT-PCR after pre-treating the ARPE-19 cells with NACA (1mM) (100μM) for 1 hour and subsequently treating with H_2_O_2_ (400μM) for 6 hours or Lactacysin for 12 hours. **(D)** ARPE-19 cells were pre-treated with NACA (1mM) for 1 hour and subsequently treated with H_2_O_2_ (200 and 400μM) for 6 hours. p62, NFκB p65 ser 536 phosphorylation, and total NFκB p65 protein levels were measured by Western blot. β-actin was used as an internal control. Representative Western blot from 3 independent experiments is shown. **(E)** ARPE-19 cells were pre-treated with MitoTEMPO (250μM) for 1 hour. Mitochondrial superoxide was measured by MitoSOX Red smitochondrial superoxide sensor. **(F)** ARPE-19 cells were either treated alone or pre-treated with MitoTEMPO (250 μM) for 1 hour and subsequently treated with H_2_O_2_ (400μM) for 6 hours or Lactacystin for 12 hours. A representative Western blot of p62 protein levels is shown. (G) ARPE-19 cells were either treated alone or pre-treated with MitoTEMPO (250 μM) for 1 hour and subsequently treated with rotenone (5 μM) for 3, 6 and 24 hours. A representative Western blot of p62 protein levels is shown.

In order to further establish oxidative stress as a key stimulator of p62 expression, we used thiol antioxidant NACA previously reported to protect RPE under oxidative stress from accumulating oxidative damage *in vitro* [37]. Pre-treatment of ARPE-19 monolayers with NACA (1mM) for one hour and subsequent challenge with H_2_O_2_ (400μM) for 6 hours resulted in dramatic significantly higher fluorescence of cellular ROS marker CM-H2DCFDA (Fig 5A). We did not see a major increase in cellular ROS when the RPE proteasome was inhibited by Lactacystin (25μM) for 12 hours. H_2_O_2_-induced p62 expression was significantly attenuated when ARPE-19 cells were pre-treated with NACA both at the protein (Fig 5B) and mRNA (Fig 5C) levels suggesting that extracellular oxidative stress plays a significant role in p62 signaling in the RPE. NACA pre-treatment, however, failed to attenuate p62 expression when ARPE-19 proteasome was inhibited with Lactacystin (Fig 5C and 5D). To further evaluate the role of oxidative stress in of NFκB S536 phosphorylation in H_2_O_2_-induced p62 expression, we subjected ARPE-19 cells to two doses of H_2_O_2_ (200 and 400 μM) for 6 hours. NFκB S536 phosphorylation was markedly reduced in NACA pre-treated cells under oxidative stress when compared to the corresponding H_2_O_2_ treated cells that were not pre-treated with NACA. We concluded that oxidative stress plays a significant role in p62 expression via modulation of NFκB activity. To determine if mitochondrial superoxide formation in RPE under oxidative stress has a role to play in stimulating p62 expression, we pre-treated the ARPE-19 cells with MitoTEMPO, a mitochondria targeted superoxide dismutase mimetic previously shown to scavenge mitochondrial superoxide [38]. As expected, challenge with H_2_O_2_ (200, 400 and 800μM) for 6 hours resulted in a dramatic increase of mitochondrial superoxide (Fig 5E). However, when ARPE-19 cells were pre-treated with MitoTEMPO, the increase in superoxide levels was significantly inhibited. Treatment with Lactacystin (25μM) for 12 hours did not show any increase in mitochondrial superoxide in either NACA pre-treated or un pre-treated ARPE-19 cells (Fig 5E). We did not observe any marked attenuation of p62 protein levels under oxidative stress or proteasomal inhibition (Fig 5F) suggesting that mitochondrial superoxide generation in RPE does not play a significant role by itself on p62 transcription. However, rotenone (a complex-1 inhibitor) that induces mitochondrial superoxide formation dramatically increased p62 levels which was attenuated by MitoTEMPO pre-treatment. This is now provided as new Fig 5G.

### p62 knockdown increases RPE cell susceptibility to oxidative stress-induced mitochondrial damage and cell death

**Fig 6:**
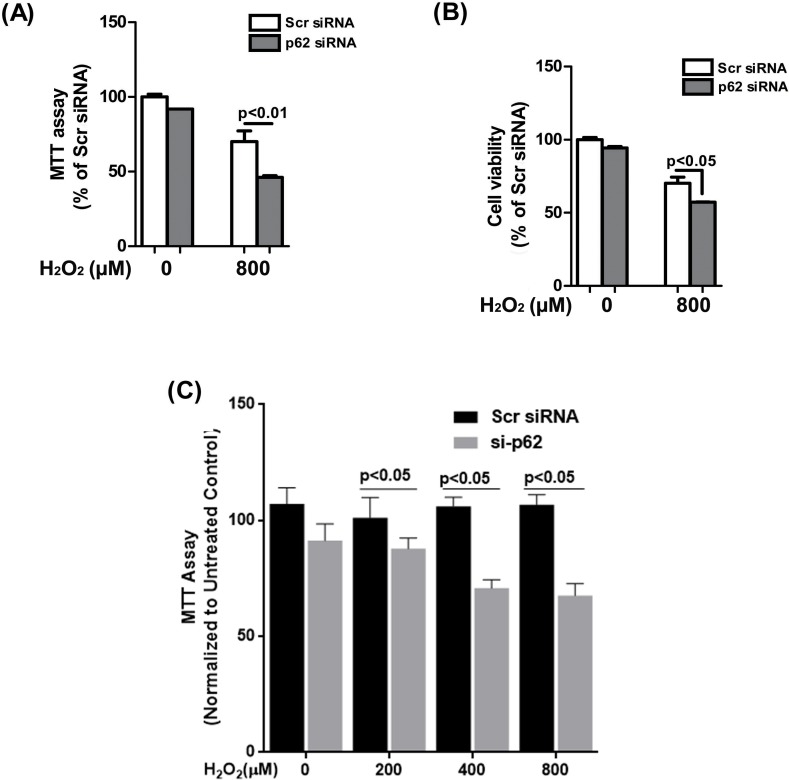
Knockdown of p62 renders RPE cells more susceptible to oxidative stress. ARPE-19 cells were transfected with p62 or scrambled siRNA for 24h and then treated with 800μM H_2_O_2_ for 6h. **(A)** Mitochondrial activity in p62 or scrambled siRNA-transfected cells was assessed using MTT assay. **(B)** Cell viability was assayed using crystal violet assay. Statistical significance was calculated by ANOVA and differences were considered statistically significant when p<0.05. **(C)** Primary fetal RPE cells were transfected with p62 or scrambled siRNA for 48h and then treated with varying concentrations of H_2_O_2_ for 24h followed by MTT assay. Statistical significance was calculated by ANOVA and differences were considered statistically significant when p<0.05.

To examine the role of p62 in oxidative stress-induced cell toxicity in the RPE, we reduced p62 expression by siRNA (85% knockdown efficiency) and tested the cellular response to a lethal dose of 800μM H_2_O_2_ for 6h. Mitochondrial activity examined by MTT assay was significantly decreased in p62 siRNA-transfected cells following H_2_O_2_ treatment compared to untreated control (Fig 6A). Loss of mitochondrial activity in ARPE-19 cell line was further corroborated by our results in primary fetal RPE (Fig 6D). We also demonstrate that H_2_O_2_-induced cell death is significantly increased in p62-silenced cell compared to scrambled control (Fig 6B). These observations show that p62 knockdown renders RPE cells more susceptible to oxidative damage.

### p62 positively regulates autophagic flux in RPE cells

**Fig 7:**
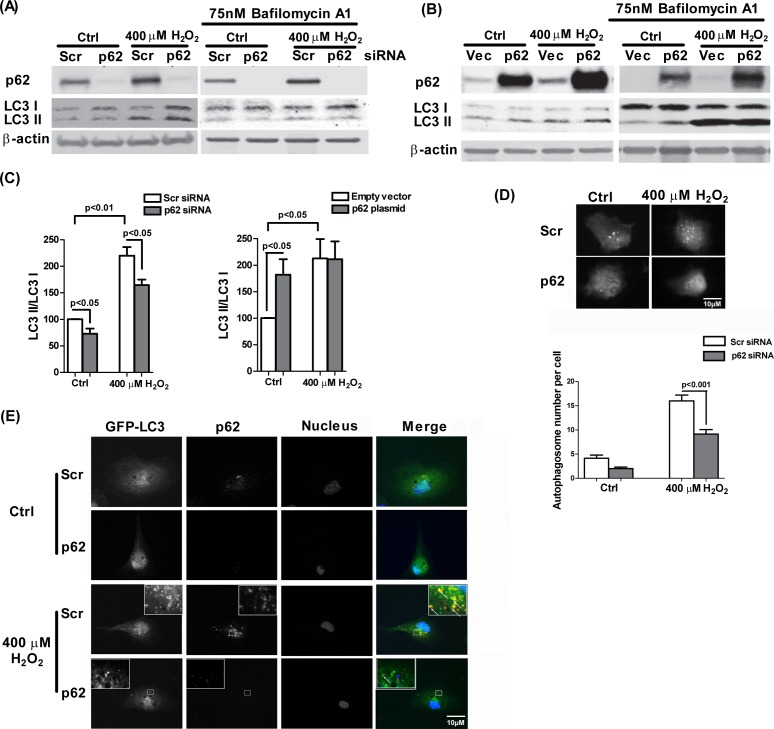
p62 positively regulates autophagic flux. LC3II/I ratio and p62 protein levels in cell lysates were examined by Western blot for **(A)** ARPE-19 cells, transfected with p62 specific siRNA or scrambled siRNA (10nM) for 24h and treated with 400μM H_2_O_2_ for additional 24 h. For assessment of autophagic flux, Bafilomycin A1 (75nM) was added 3h prior to harvesting in one set of treatments per experiment. **(B)** RPE cells transfected with p62 construct or empty vector for 24h and treated with 400μM H_2_O_2_ for an additional 24 h. β-actin was used as an internal control. **(C)** Densitometric analyses of LC3 II/I ratio in (A = endogenous p62 knock-down and B = ectopic overexpression of p62) were plotted as mean + S.E.M. of three independent experiments. Mean differences were considered statistically significant when p<0.05 by Mann-Whitney U-test. **(D)** Autophagosome numbers were determined in RPE cells co-transfected with GFP-LC3 and p62 siRNA or scrambled siRNA (10nM), after H_2_O_2_ treatment. 50 cells per condition per experiment were analyzed for autophagosome numbers. Differences between mean counts from 3 independent experiments were considered statistically significant when p<0.05 by Mann-Whitney U-test. **(E)** RPE cells transfected with GFP-LC3 together with p62 siRNA or scrambled siRNA for 24h and treated with 400μM H_2_O_2_ for additional 24h were fixed and immunostained for p62. AlexaFluor^®^ 594-conjugated goat anti-mouse secondary antibody was used for visualization. White arrows represent the co-localization of p62 (red) and GFP-LC3 (green).

Autophagic degradation offers an alternative protein degradation pathway to UPS and is likely to be activated when the UPS is overloaded with substrate. To further test the involvement of p62 in autophagic regulation in the RPE, p62 was knocked down by siRNA or overexpressed by p62 plasmid transfection. The efficacy of p62 siRNA or expression level of p62 construct was then analyzed. Western blot analysis revealed effective blocking or increase of the p62 synthesis in response to the mRNA silencing or plasmid transfection respectively (Fig 7A and 7B). Autophagy was examined by LC3 lipidation and autophagosome formation in the presence or absence of 400μM H_2_O_2_ with or without p62 silencing/overexpression for 24 h. In the presence or absence of external oxidative stress, knockdown of p62 decreased the ratio of LC3 II/I. In contrast, p62 overexpression significantly enhanced the ratio of LC3 II/I ratio in the absence of H_2_O_2_ implying an increase in autophagic flux, and peroxide treatment did not affect the ratio when p62 level was elevated (Fig 7C). Changes in autophagy dynamics were confirmed by assessing the formation of autophagosome puncta containing GFP-LC3. The number of fluorescent punctae significantly increased in scrambled siRNA-transfected cells after H_2_O_2_ treatment but were greatly reduced in cells treated with p62 siRNA (Fig 7D). Furthermore, confocal microscopy revealed greater association of p62 with GFP positive LC3 punctate dots (Fig 7E) following H_2_O_2_ exposure supporting active association of p62 in the autophagic process under oxidative stress.

### ATG10 is associated with p62-regulated autophagy

**Fig 8:**
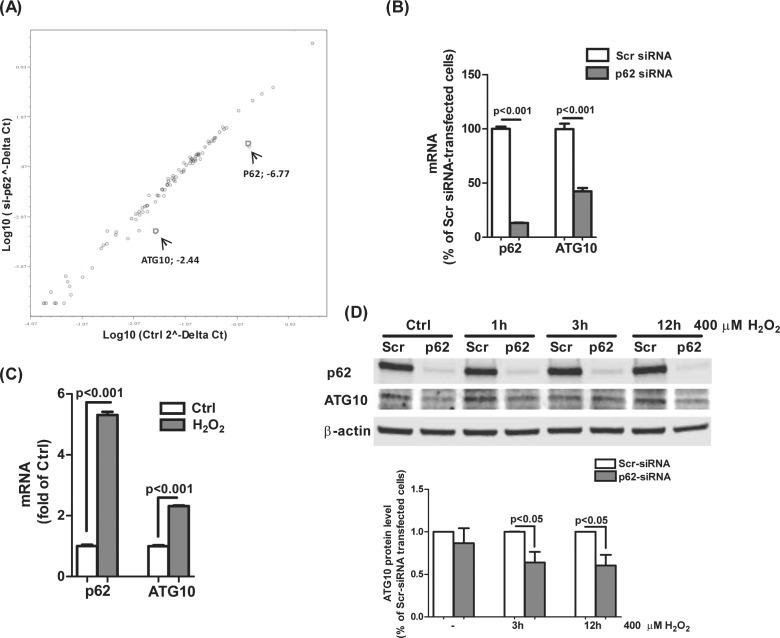
ATG10 is involved in p62-regulated autophagy. **(A)** Scatter plots comparing the normalized, relative expression of autophagy-related proteins in ARPE-19 cells transfected with p62 specific or scrambled siRNA (10nM) for 24 h. mRNA expression was detected using autophagy qRT-PCR array from SA Biosciences each. The 'fold-change boundary' denoted by the parallel lines flanking the median line was set at 2 and segregates the genes up or down regulated. Dots represent genes plotted on the basis of Log _10_(2^-ΔΔCt^). Blue circles denote the genes ATG10 and p62 with -2.44 and -6.77 fold changes respectively. **(B)** p62 mRNA level was examined by qRT-PCR to confirm the knockdown of p62 transcription and mRNA level of autophagic protein ATG10 was assessed in p62-knockdown cells. **(C)** RPE cells were treated by 400μM H_2_O_2_ and ATG10 mRNA level was measured by qRT-PCR. Mean+ S.E.M. from three independent experiments were plotted. **(D)** RPE cells were transfected with p62 or scrambled siRNA for 24h and then exposed with 400 H_2_O_2_ for 1, 3, 6, 12h respectively. Whole-cells lysates were analyzed by Western blot with anti-p62, ATG10, or β–actin antibodies. Differences between means were considered statistically significant when p<0.05 by Mann-Whitney U-test.

Having shown that p62 is associated with autophagosomes and positively regulates the autophagic process in RPE cells (Fig 8) we screened an autophagy qRT-PCR array to identify which transcripts are regulated by p62. The mRNA levels of the ATG proteins on the array were compared between untreated control and p62-silenced cells in the absence of external oxidative stressor. Several autophagy protein transcripts were up or down regulated by silencing p62 (Table 1) of which ATG10, an E2-like enzyme involved in autophagy, showed a large decrease (>2 fold) in expression in p62-knockdown cells (Fig 8A). ATG10 primer sets were then used to validate the results from qRT-PCR array and demonstrated that ATG10 expression decreased by over 50% in p62-deficient cells (Fig 8B). ATG10 expression was increased 2 fold in H_2_O_2_-treated cells compared to control as was p62 expression which acted as a positive control (Fig 8C). Furthermore, ATG10 protein level also showed a significant decrease in p62-knockdown cells (Fig 8D).

**Table 1:** Differentially expressed genes after p62 knockdown.

Gene Name	Fold Change
ATG10 autophagy related 10 homolog (ATG10)	-2.44
Sequestosome 1/p62	-6.77
Interferon, gamma (IFNG)	-1.93
Tumor Necrosis Factor (TNF)	-1.89
Interferon, alpha 4 (IFNA4)	-1.93
Tumor protein p73(TP73)	-1.78
Phosphoinositide-3-kinase, catalytic, gamma polypeptide (PIK3CG)	-1.86

### ATG10 deficiency renders RPE more susceptible to oxidative stress

**Fig 9:**
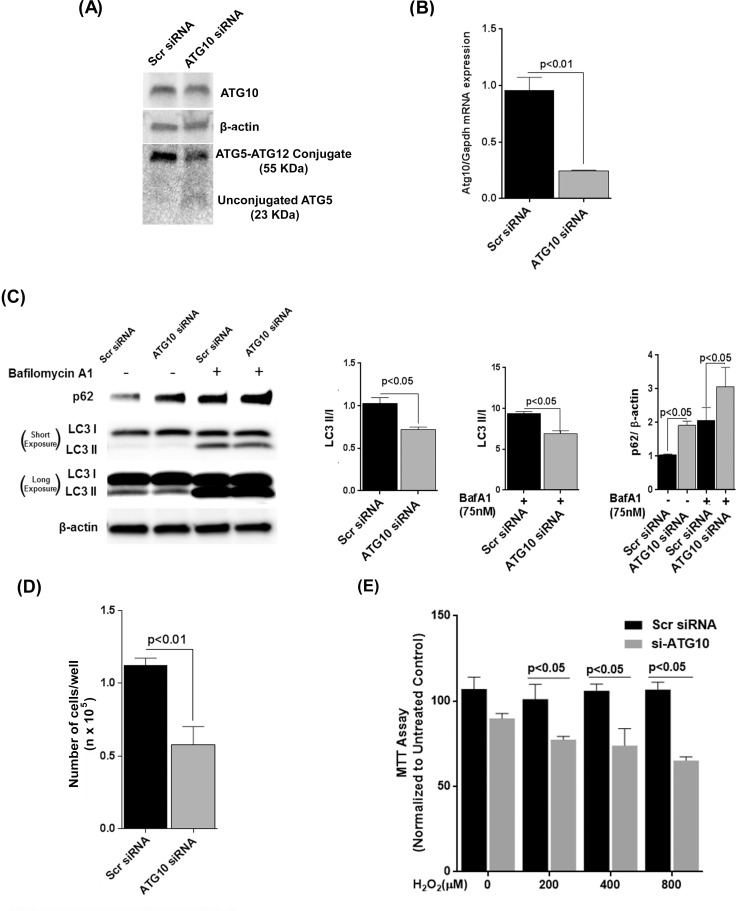
Knockdown of ATG10 makes ARPE-19 cells more sensitive to H_2_O_2_. **(A)** ARPE-19 cells were transfected with pooled ATG10 specific siRNAs or scrambled siRNA (30nM) for 24h. ATG10 and ATG5 levels were assessed in protein lysates by Western Blot. **(B)** RNA isolated from cells was analyzed for ATG10 mRNA expression by qRT- PCR. Differences between means were considered statistically significant when p<0.05 by Mann-Whitney U-test. **(C)** Lysates of ARPE-19 cells transfected with siRNAs for 24 hours were assessed for p62 and LC3 levels by Western blot. One set of wells per experiment was exposed to Bafilomycin A1 (75nM) for 3h prior to harvest. β-actin was used as an internal control. ‘Short’ and ‘Long’ Exposure indicate time of exposure of immunoblot image. **(D)** 24h after transfection with respective siRNAs, ARPE-19 were subjected to oxidative stress with 800μM H_2_O_2_ and incubated at 37°C for 6h. Total cell numbers were determined by trypsinization and counting on TC10^®^ cell counter. **(E)** Primary fetal RPE cells were transfected with scrambled or pooled ATG10 specific siRNAs and 48 hours after transfection, were subjected to H_2_O_2_ for 24 hours and MTT assay was performed. Differences between means were considered statistically significant when p<0.05 by Student’s t-test.

To substantiate the importance of ATG10 in the RPE we transiently knocked ATG10 down in ARPE-19 cells (Fig 9A and 9B). 24h after ATG10 was knocked down, ARPE-19 cells showed an increased amount of unconjugated ATG5 (23KDa) reduction in ATG5-12 (55KDa) conjugate level (Fig 9A). A lower amount of autophagic activity was observed as measured by LC3 II/I ratio in either the presence or absence of Bafilomycin A1 (Fig 8C). ARPE-19 cells with reduced ATG10 expression exhibited significant loss of cell numbers when challenged by 800μM H_2_O_2_ (Fig 9D). Fetal RPE cells showed significant loss in mitochondrial activity 24 hours after being challenged by varying concentration of H_2_O_2_ (200, 400 and 800μM) (Fig 9E). This mimicked the results observed for p62-knockdown cells in response to oxidative stress suggesting that the increase in susceptibility to oxidative stress for RPE cells when p62 expression is reduced could be attributed to autophagic deficiency involving loss of ATG10 expression.

## Discussion

A growing body of evidence from cell culture and animal models suggests that exposure to oxidative stress is a key mediator of RPE injury and an important cause of AMD (45,47,48). Recent studies from our laboratory demonstrated that autophagy is a critical regulator in the RPE and plays an important role in protection against oxidative stress and lipofuscin accumulation [9, 30]. In the present study, we report that p62 is dramatically up-regulated under oxidative stress, which is associated with enhanced autophagic flux in RPE cells and that p62 silencing renders RPE cells more susceptible to oxidative stress. Our results revealed that oxidative stress-induced proteasome inhibition is a trigger to induce p62-assisted selective autophagy to remove protein aggregates and damaged organelles since oxidative exposure blocked proteasome activity; consistently, we demonstrated that proteasome specific inhibitor Lactacystin increases both p62 and autophagic flux. Our observation that lactacystin up-regulates p62 is consistent with previous reports that proteasome inhibitor MG-132 caused increased p62 cellular immunostaining and perinuclear accumulation in human RPE cells [18, 39].

Our data demonstrates that oxidative stress induces p62 gene expression in the RPE, which activates the autophagic pathway and serves as an early protective response against oxidative stress. This compliments the finding by Wang *et al*. that shows that isoform 1 of p62 is up-regulated selectively by cigarette smoke-induced stress as a cytoprotective response [40]. These authors showed, using a different cellular stressor, that p62 silencing exacerbated accumulation of damaged proteins both by suppressing autophagy and by inhibiting the Nrf2 antioxidant response. While p62 has been suggested to be an immediate early response gene in fibroblasts, lymphocytes and promyelocytes in reaction to antigenic stimulatory agents [32], we show that p62 activation is an early response critical for RPE survival and homeostasis via the autophagic pathway against a general oxidative stressor (H_2_O_2_). Our data, taken together with the findings by Wang *et al*., reinforce the critical role of p62 in RPE autophagy [40].

To assess the specific role of p62 on RPE autophagy, we then examined the regulatory function of p62 on autophagic gene expression and observed ATG10 showed a major reduction. ATG10 is an E2-conjugating enzyme catalyzing autophagic vacuole formation including ATG5-ATG12 conjugate formation and LC3 lipidation [41–43]. Autophagic vacuole formation is the fundamental step to initiate autophagy and this report is the first to show that p62 regulates autophagosome biogenesis and autophagic flux through regulating autophagy machinery proteins, such as ATG10 in RPE cells. To further establish the importance of ATG10 in RPE cells, we show that transient attenuation of endogenous ATG10 expression increases the susceptibility of RPE to oxidative stress.

As a protein selectively degraded by autophagy, p62 has been used as a general indicator for autophagic flux: p62 accumulation indicates the autophagy deficiency whereas p62 reduction suggests a high-rate of autophagic process. Previous studies demonstrated that the foveomacular areas of AMD patients show increased p62 staining compared to the perimacular and peripheral areas [39]. The same does not hold true for the age-matched control donors suggesting that autophagic degradation of p62 may be compromised in diseased RPE [39]. Our observations in a rodent model of AMD corroborate this finding. However, we have to point out that p62 is a multifunctional factor involved in several cellular pathways and its protein level is regulated not only by degradation but also by gene expression independent of autophagy [44]. For example, a recent study in RPE cells demonstrated that hydroxytyrosol (HT), a natural phytochemical from olive leaves and oil increased p62 mRNA and protein level but this phenomenon did not correlate with abolishment of autophagy [45]. HT stimulates the antioxidant response via Nrf2, suggesting that it stimulates p62 transcription via the ARE in the p62 promoter [46]. Consistent to this, our *in vitro* data demonstrates that oxidative stress in RPE enhanced autophagic flux in conjunction with increased p62 protein level. Therefore, p62 protein level may not be a reliable marker for autophagy in certain circumstances. We thus recommend examining both mRNA level and protein level of p62 before making any conclusion of autophagy activation or inactivation based on p62 data. Combining these observations, we conclude that the degradation pathways of p62 in AMD are compromised.

p62, an adaptor protein with a number different domains, can bind to several critical signaling factors, such as atypical PKCs, the signaling adapter RIP, the E3 ubiquitin ligase TRAF6, the kinase ERK, caspase 8 and Keap1 (19,45,55,56) and thereby activate various cell signaling pathways [47]. In fact, p62 has been shown to promote the Nrf2-mediated antioxidant response in the mouse liver [20] a finding recently corroborated in the RPE [40, 48]. However, recent reports suggest that NFκB signaling and p62 expression are intimately involved in defining cellular fate. NFκB signaling is a key player in several cellular responses and plays a major role in cellular survival under stress. Our results demonstrated cytoprotective effect of p62 in RPE under oxidative stress. Although the p62-Nrf2 signaling pathway has previously been established to play a cytoprotective role in the RPE[40], we wanted to determine if NFκB is involved in p62 upregulation. NFκB activation requires dissociation from IκB and subsequent p65 phosphorylation at serine residues 276, 529, and 536 to facilitate nuclear translocation [49]. We observed transient activation (by Serine 536 phosphorylation) and nuclear translocation of NFκB in RPE under H_2_O_2_-induced oxidative stress. The activation (i.e. Serine 536 phosphorylation) was maximum at 3 hours post-challenge concomitant with the induction of p62 mRNA. Furthermore, pharmacologically inhibiting IKKβ by SC-514, which mediates dissociation of NFκB from IκB and subsequent activation, resulted in significant attenuation of p62 expression. This was accompanied by a loss of LC3 (II/I) ratio compared to control cells (untreated by SC-514) suggesting that autophagy was diminished when NFκB activation was inhibited. NFκB Ser 529, 536 short peptide described by Takada *et al*. is a competitive inhibitor for p65 subunit phosphorylation (59). Incubation of RPE cells with the Ser529, 536 short peptide showed significant reduction of p65 Serine 536 phosphorylation 3 hours post H_2_O_2_-challenge and resulted in attenuation of p62 response. These results suggest that in RPE under oxidative stress, NFκB may play a key role in regulating p62 expression and subsequently stimulating autophagy. One of the key aspects of NFκB activation is the proteasomal degradation of the IκB that dissociates from the complex upon phosphorylation by IKK (reviewed in [50]). If that were the case for RPE cells, then proteasomal inhibition by Lactacystin would result in deactivation of NFκB signaling. However, in our experiments, Lactacystin incubation for 12 hours showed the exact opposite result with dramatic increase in p65 Serine 536 phosphorylation with concomitant increase in p62 expression. This apparently paradoxical observation of sustained NFκB activation when proteasomal degradation is inhibited has been recently reported in brain glial cells [51]. Although initially Lactacystin reduces both IκB-α and IκB-β levels, at later timepoints, it seems to upregulate unphosphorylated IκB-β preferentially. NFκB bound to unphosphorylated IκB-β is still capable of nuclear translocation [51]. This may explain the increase of p65 Serine 536 phosphorylation in the RPE when treated with Lactacystin. The role of augmented NFκB signaling when proteasome is inhibited in RPE is also strengthened by our observations that antioxidant treatment with NACA fails to attenuate Lactacystin induced p62 expression while H_2_O_2_ induced p62 expression can be sufficiently attenuated although H_2_O2 –challenge itself can impair the proteasome. This suggests that oxidative stress may upregulate p62 expression either by the well-established Nrf2 signaling pathway or by augmenting NFκB signaling because of proteasome inhibition.

## Conclusions

We establish that p62/SQSTM1, a critical factor involved in both UPS and ALP degradation systems, was dramatically up-regulated in the RPE at both mRNA and protein level following exposure to oxidative stress and that this was, at least in part, mediated by NFκB. Our results in the mouse model supported the previously reported observation of increased p62 in human AMD patient retinas. Furthermore, we demonstrated that amplifying p62 expression increases autophagy and protects RPE cells from oxidative exposure-induced cell death and downregulation of p62 reduces autophagical degradation and make RPE cells more vulnerable to oxidative treatment. More importantly, for the first time, we show that p62 modulates autophagy by regulating expression of ATG10. Understanding the role of oxidative stress-induced stimulation of p62 in the RPE cells will provide new information on the pathogenesis of AMD. Furthermore, we strongly believe that a detailed clarification of the signaling pathways and function of p62 in autophagic degradation pathway, which plays a pivotal role in RPE cells, may enable development of specific drugs for the treatment of AMD.
